# Effectiveness of perampanel in managing chronic pain caused by the complex regional pain syndrome: A case report

**DOI:** 10.1097/MD.0000000000027791

**Published:** 2021-12-03

**Authors:** Min Cheol Chang, Donghwi Park

**Affiliations:** a Department of Rehabilitation Medicine, College of Medicine, Yeungnam University, Daegu, Republic of Korea; b Department of Physical Medicine and Rehabilitation, Ulsan University Hospital, University of Ulsan College of Medicine, Ulsan, Republic of Korea.

**Keywords:** α-amino-3-hydroxy-5-methyl-4-isoxazole propionate, chronic pain, complex regional pain syndrome, medication, treatment

## Abstract

**Rationale:**

The α-amino-3-hydroxy-5-methy-4-isoxazole propionate (AMPA) receptor plays a critical role in the development and persistence of pain, and AMPA receptor antagonists are considered possible therapeutic targets for controlling pain. This report describes a patient with complex regional pain syndrome (CRPS) type I in the right lower leg and foot who responded well to perampanel, an AMPA receptor antagonist, for managing the chronic pain.

**Patient concern:**

A 61-year-old woman complained of pain in her right lower leg and foot over a period of 7 year (numeric rating scale: 8) due to CRPS type I.

**Diagnosis:**

CRPS type 1.

**Interventions:**

Despite the combination of 300 mg pregabalin, 225 mg/1950 mg tramadol/acetaminophen, and 10 mg nortriptyline per day, her right lower leg and foot were nearly disabled due to the severity of the pain. High-dose prednisolone was found to be ineffective. Then, perampanel (4 mg; 2 mg twice) was administered to this patient daily.

**Outcomes:**

The day after treatment with perampanel, her pain completely disappeared. Additionally, at day 7 and 1 month follow-up, she reported no pain in the right lower leg and foot. Moreover, no adverse effects were reported after the application of perampanel.

**Lessons:**

These results suggest that perampanel may potentially be used to treat centralized pain.

## 1 Introduction

The α-amino-3-hydroxy-5-methyl-4-isoxazole propionate (AMPA) receptors play critical roles in the mechanisms of acute and chronic pain.^[1]^ AMPA receptor-expressing neurons in the spinal dorsal horn receive primary afferent nociceptive inputs.^[1]^ It is a receptor for glutamate, which is the most important neurotransmitter of excitatory synapses.^[2]^ Upon blinding to glutamate, AMPA receptors activate the N-methyl-D-aspartate receptors and allow an influx of Ca^2+^ ions.^[3,4]^ Then, Ca^2+^ influx activates a series of downstream signaling proteins, such as kinase and other transporter proteins, and recruits more AMPA receptors to the synapse.^[3,4]^ This process strengthens the synaptic transmission from peripheral to spinal neurons, which underlies the basic mechanism of central sensitization. Central sensitization is defined as the amplification of neural signaling within the central nervous system that elicits pain hypersensitivity.^[5]^

Blockage of the AMPA receptor has been reported to alter pain sensitivity in animal studies and is considered to be a possible therapeutic target for controlling pain.^[6–9]^ To date, several AMPA receptor antagonists have been developed and their positive pain-reducing effects have been reported in many animal studies^[6–9]^; however, little is known about their effects in humans.

In the current study, we report successful pain management using perampanel, an AMPA receptor antagonist, for a patient with complex regional pain syndrome (CRPS) type I.

## 2 Case report

A 61-year-old woman visited the Physical Medicine and Rehabilitation Department of a university hospital due to pain in her entire right lower leg and foot over a period of 7 year (numeric rating scale: 8; range: 0–10; 0: no pain, 10: worst pain imaginable), which was aggravated by standing and walking (numeric rating scale: 9). Her right lower leg and foot were nearly disabled due to the severity of the pain. The patient provided informed consent for participation in the study. The study was approved by the local institutional review board of Yeungnam University hospital. Previously, she had suffered a fracture of her right metatarsal bone and underwent an open reduction and internal fixation surgery to manage the pain; however, the pain persisted even after this pain removal operation. On physical examination, she was found to have hyperesthesia and allodynia of her entire right lower leg and foot. Skin atrophy and hypohidrosis were observed in the left lower leg and foot. She also said that her right lower leg and foot were edematous and reddish for approximately 2 year after the initiation of pain. In a nerve conduction study and lumbar spine magnetic resonance imaging, no abnormal findings were noted. Based on the CRPS criteria proposed by the Budapest consensus group,^[10]^ we diagnosed the patient with CRPS type I. In the local pain clinic, she was receiving treatment with oral medications, including 300 mg pregabalin (150 mg, twice), 225 mg/1950 mg tramadol/acetaminophen (75 mg/650 mg, thrice), and 10 mg nortriptyline (10 mg, once) per day. However, only a minimal pain-reducing effect was observed. Initially, along with these medications, we administered 60 mg of prednisolone per day; however, 7 day after initiating the treatment with prednisolone, no pain reduction was observed. Moreover, she complained of epigastric pain and insomnia; therefore, we stopped the treatment with prednisolone and initiated the administration of 4 mg perampanel (2 mg, twice) daily. The day after treatment with perampanel, her pain completely disappeared. Moreover, at the 7 day and 1 month follow-up, she reported no pain in the right lower leg and foot. Pain was not observed even during standing and walking. Additionally, no adverse effects were reported after perampanel application.

## 3 Discussion

We successfully controlled the chronic pain from CRPS using a selective AMPA receptor antagonist, perampanel, administered at 4 mg per day. We hypothesized that AMPA receptor antagonists may inhibit the synaptic transmission of centralized pain signals from the peripheral to spinal neurons as well as the amplification of pain signals within the spinal cord. This mechanism of AMPA receptor antagonist seems to be attributed to the reduction of CRPS pain in our patient.

To date, several animal studies have demonstrated the pain-reducing effects of AMPA receptor antagonists.^[6–9]^ However, to the best of our knowledge, only 4 previous studies have evaluated the pain-reducing effects of AMPA receptor antagonists in humans.^[11–14]^ In 1998, Sang et al.^[12]^ performed a preliminary study to evaluate the effect of the AMPA receptor antagonist, LY293558. They injected capsaicin intradermally to evoke hyperalgesia in 15 healthy volunteers, compared the effect of intravenous LY293558 infusion with that of placebo, and followed-up the effect for 1 hours. They found significantly reduced pain intensity and unpleasantness after the infusion of LY293558. However, most volunteers reported the side effects of hazy vision or sedation. In 2004, Sang et al.^[13]^ evaluated the effect of LY293558 on migraines. The response rate was 69% after the intravenous administration of LY293558. It was higher than the placebo treatment (25%), but lower than that of the sumatriptan treatment (86%). In this study, 2 out of 13 patients reported side effects, including visual symptoms and sedation. In 2009, Gormsen et al^[11]^ treated 13 patients with chronic neuropathic pain due to peripheral nerve injury using the intravenous AMPA receptor antagonist, NS1209, and found significant pain reduction in the placebo group. However, only 20% to 30% of the patients showed a moderate or good response to the treatment. Of the 13 patients, 8 exhibited side effects, such as headache, dizziness, somnolence, feeling abnormal, fatigue, dry mouth, and nausea. In 2012, Wallace et al^[14]^ recruited 18 subjects and conducted a crossover study. They injected capsaicin into the volar region of the forearm to evoke hyperalgesia. The included subjects received 150 mg or 90 mg of the AMPA receptor antagonist, NGX426, or placebo orally. The authors found that pain was significantly reduced after the application of NGX426, and 150 mg NGX426 was more effective than 90 mg NGX426. The administration of 150 mg NGX426 led to somnolence in 3 subjects, dizziness in 1 subject, and light-headedness in 1 subject. All of these previous studies were conducted with drugs that were not released as products in the market. Although these drugs showed good pain reduction effects, various side effects were observed in many subjects.

Perampanel is a selective and non-competitive AMPA receptor antagonist that is used as an antiepileptic drug in clinical practice.^[15]^ In addition, perampanel is suggested to have a pain-reducing effect by inhibiting the Ca^2+^ influx into spinal neurons and subsequently hindering the activation of downstream signaling proteins. To evaluate the pain reduction effects of perampanel and its related mechanisms, various animal studies were conducted.^[16–18]^ Khangura et al^[17]^ performed an animal study in rats and found that neuropathic pain induced by chronic constriction injury was attenuated by the oral administration of perampanel (3 and 6 mg/kg), while naloxone significantly decreased the pain-attenuating effects of perampanel. Based on this finding, they suggested that perampanel exhibits an antinociceptive effect by acting on the opioid system. Moreover, Tringali et al^[18]^ found that perampanel inhibited the release of calcitonin gene-related peptides. Our patient's chronic pain from CRPS seems to have been reduced via these mechanisms.

When 4 mg of perampanel was applied, about 30% of the patients reported side effects, such as dizziness, somnolence, headache, fatigue, and vertigo.^[19]^ Due to the high possibility of the occurrence of side effects, clinicians should have knowledge of the possible side effects of perampanel and pay attention to their occurrence during the treatment with perampanel.

In conclusion, we described the successful treatment of chronic pain in a patient with CRPS type I using perampanel (4 mg/day). Our report suggests that perampanel might be a useful drug to control the refractory centralized pain. To the best of our knowledge, this is the first study to show the positive pain-reducing effects of perampanel or commercialized AMPA receptor antagonists in humans. However, our study is limited in that it was a case report. Further studies with a large number of cases are needed to elucidate the effects of perampanel.

## Author contributions

**Writing – original draft:** Min Cheol Chang, Donghwi Park.

**Writing – review & editing:** Min Cheol Chang, Donghwi Park.
